# WHO should accelerate, not stall, rectal artesunate deployment for pre-referral treatment of severe malaria

**DOI:** 10.1093/trstmh/trad002

**Published:** 2023-02-01

**Authors:** Thomas J Peto, James A Watson, Nicholas J White, Arjen M Dondorp

**Affiliations:** Mahidol Oxford Tropical Medicine Research Unit, Faculty of Tropical Medicine, Mahidol University, Bangkok, Thailand; Centre for Global Health and Tropical Medicine, Nuffield Department of Medicine, University of Oxford, UK; Mahidol Oxford Tropical Medicine Research Unit, Faculty of Tropical Medicine, Mahidol University, Bangkok, Thailand; Centre for Global Health and Tropical Medicine, Nuffield Department of Medicine, University of Oxford, UK; Mahidol Oxford Tropical Medicine Research Unit, Faculty of Tropical Medicine, Mahidol University, Bangkok, Thailand; Centre for Global Health and Tropical Medicine, Nuffield Department of Medicine, University of Oxford, UK; Mahidol Oxford Tropical Medicine Research Unit, Faculty of Tropical Medicine, Mahidol University, Bangkok, Thailand; Centre for Global Health and Tropical Medicine, Nuffield Department of Medicine, University of Oxford, UK

## Abstract

The recent World Health Organization moratorium on rectal artesunate (RAS) for pre-referral treatment of severe childhood malaria is costing young lives. The decision was based on disappointing findings from a large observational study that provided RAS to community health workers with little training and supervision. This non-randomized, operational research has provided useful information to guide the implementation of RAS but is subject to bias and confounding and cannot be used to assess treatment effects. Parenteral artesunate reduces severe malaria mortality and a large body of evidence also shows RAS has lifesaving efficacy. There is now more than a decade of delay in conducting the necessary engagement and training required for successful deployment of RAS. Further delays will result in more preventable deaths.

Nobody would wish to leave a child with severe malaria untreated for hours or days until they can reach a healthcare facility where parenteral antimalarial treatment is available. Rectal artesunate (RAS) allows early treatment to be given in or near the home. Its lifesaving efficacy was demonstrated in a very large randomized controlled trial (RCT).^1^ For drugs proven to be efficacious in well-conducted RCTs, evidence from operational research can usefully identify important real-world challenges to their deployment. Efficacy in trials does not equate directly to effectiveness in real life, particularly in resource-limited settings in the tropics.^2^ Yet operational studies are an unreliable source of evidence from which to assess the direct causal effects of treatment. Well-conducted randomization largely avoids the biases that confound observational studies.^3^ The recent change in World Health Organization (WHO) recommendations on RAS for the initial community management of children with suspected severe malaria is a graphic and costly illustration of this problem. Despite a substantial evidence base for the lifesaving efficacy and excellent safety of artesunate in severe malaria derived from RCTs, the WHO has advised a moratorium on the deployment of RAS based on heterogeneous and disappointing results from a sequential observational study that was partially disrupted by the SARS-CoV-2 pandemic.^4^ The correct response to these results would be to recognize deployment problems and focus on how to improve them rather than the scientifically unsound and ethically questionable recommendation that has resulted in withholding of a potentially lifesaving drug from severely ill children.

There is still no satisfactory strategy to address the unacceptably high numbers of African children who die from malaria in rural areas.^5^*Plasmodium falciparum* malaria can progress rapidly to severe disease and death. In severe disease, parenteral antimalarials save lives but, at the community level, injectable treatment is commonly unavailable. In the early 2000s, RAS was shown to be an effective treatment of *P. falciparum* malaria in hospitalized patients and, when given as a pre-referral treatment for suspected severe malaria, in large community-based trials.^1,6^ The mortality benefit from RAS was among patients with a substantial delay in accessing injectable treatment, which in the phase 3 trial was defined as those not in hospital within 6 h.

Based on the clinical evidence, RAS is recommended in the WHO treatment guidelines and appears in the treatment guidelines of many malaria-endemic African countries. Quality-assured RAS was prequalified in 2018 (a full decade after the phase 3 trial was reported) and approximately 3 million doses were supplied to 20 countries by the end of 2020,^7^ although it is unclear to what extent RAS has actually reached rural community health workers and their patients. Although RAS is inexpensive, safe, well-tolerated, easy to administer and can be given by community health workers, it has not been implemented at sufficient scale and so has not made a significant impact on malaria mortality. There are many reasons for this, including delays in manufacturing, guideline development and the drug registration process itself—some of which are understandable—although why, with all the organizations and resources devoted to malaria, this has been so slow (>10 y) is concerning.

A central issue is the need for reliable follow-up antimalarial treatment after rectal artesunate, ideally with parenteral artesunate and a full course of an artemisinin-based combination therapy (ACT). Over the past decade, operational research activities in several African settings provided RAS to febrile children in villages who could not reliably take oral antimalarial medicines (i.e. suspected severe malaria). The largest implementation study of RAS was the Community Access to Rectal Artesunate for Malaria (CARAMAL) study in Nigeria, Uganda and the Democratic Republic of Congo. It was the disappointing results of this study that led directly to WHO's decision to halt deployment of RAS. The CARAMAL study was interpreted as showing that RAS deployment was associated with worse referral patterns and that this resulted in increased case fatality ratios. It was hypothesized that parents or caregivers were reassured by the administration of RAS and delayed referral of the sick children—with fatal consequences.^8,9^ However, the study does not provide reliable evidence of either of these effects and there are serious concerns over the causal interpretation of this study.^10^ The CARAMAL study does not exclude major confounders (such as community health workers preferentially giving RAS to the sickest children) or other explanations (such as sepsis rather than malaria being the main cause of death).^10^ The contrast of the observational study of RAS deployment in Nigeria, which claimed an implausibly large increase in mortality, most of which was within 48 h of RAS administration, to another observational study in Zambia, which claimed an implausibly large decrease in mortality, highlights the unreliability of using such study designs to estimate treatment effectiveness.^9,11^ A separate concern over RAS deployment is that it will increase drug pressure to select resistance from artesunate monotherapy. However, RAS is very unlikely to be a significant driver when compared with other sources of resistance.^12^ This is not a reason to withhold a potentially lifesaving treatment from a sick child.

The WHO's moratorium on the use of RAS following the CARAMAL study has had very damaging consequences: once discontinued, it becomes difficult to redeploy RAS when the trust of the national malaria control programmes and healthcare workers has been undermined. So how best should RAS be used when this ill-judged moratorium is reversed? The CARAMAL study reported that RAS was well-accepted by healthcare providers and caregivers at all sites and that most children with suspected severe malaria who attended a community health worker went on to receive care from a secondary provider, although not generally from a referral hospital. The authors concluded that ‘alternative effective treatment options should be provided to children unable to complete referral’.^8^ This implies RAS deployment should be part of serious efforts to improve referral, expand access to early treatment with effective oral antimalarial drugs and provide parenteral consolidation treatment at the most peripheral level of the health system that is practicable. In other studies, it was possible to achieve substantial improvements to referral with limited additional resources.^13^ Therefore there is a need during the deployment of RAS to identify barriers to access and referral and find locally appropriate solutions. Case management algorithms incorporating RAS need to reflect local reality and be part of strengthening of existing health systems, chiefly via training and supervision. But to withhold RAS deployment where referral cannot be guaranteed would be a mistake. RAS is likely to have the greatest impact where health services are weakest.

Early treatment of severe malaria will reduce childhood mortality in rural Africa. Artesunate is the best available treatment for life-threatening *P. falciparum* malaria. The training and support mechanisms for community health workers to use RAS appropriately and effectively are important, so the correct approach, if they are inadequate, is to strengthen them—not to withdraw RAS. Preventing children with potentially life-threatening malaria from receiving an effective treatment is wrong. The WHO moratorium on RAS should be lifted.
